# Determining the influence of fava bean pre-processing on extractability and functional quality of protein isolates

**DOI:** 10.1016/j.fochx.2024.101200

**Published:** 2024-02-08

**Authors:** Mohammad Hassan Kamani, Jianlei Liu, Sinead M. Fitzsimons, Mark A. Fenelon, Eoin G. Murphy

**Affiliations:** a Food Chemistry and Technology Department, Teagasc Food Research Centre, Moorepark, Fermoy, County Cork, Ireland; b Academy of National Food and Strategic Reserves Administration, Beijing 102629, China

**Keywords:** Fava bean, Dehulling, Protein isolate, Protein extraction, Milling

## Abstract

In this study, fava bean protein (FPI) was isolated from flours prepared from dehulled seeds and compared to FPI extracted from whole flours; in the latter case, flours were prepared either by dry- or wet-milling. Significant differences in composition and functionality were observed between the three FPIs produced. Dehulling maximized protein purity, oil-absorption capacity, solubility, foamablity and minimized both starchy and non-starchy carbohydrate contents. Protein isolated from wet-milled whole beans provided higher mass and extraction yields, better water-absorption capacity, and exhibited higher surface charge (zeta potential) compared to other samples. The protein extracted from dry-milled whole seed exhibited a higher least gelation concentration, emulsifying activity and zeta value compared to its dehulled counterpart. Dehulling was also found to be a useful process to increase the lightness/whiteness of protein powder. Overall, the present findings provide useful technological information relating to the production of FPI with and without a dehulling step.

## Highlights

• Pre-processing influenced extractability and functionality of fava bean protein.
• Dehulling is an important step to produce protein with high purity and solubility.
• Soaking of beans prior to wet milling led to increased protein yield and WAC.

## 1 Introduction

Legumes represent a healthy and inexpensive protein source for humans, and their use is established for industrial food applications (Sharan et al., 2021, Boukid and Castellari, 2022). One such legume is fava bean (*Vicia faba* L.) which in recent years has received increased attention, typically in grain (pulse) form, as a source for protein extraction (Boukid and Castellari, 2022, Bangar and Dhull, 2022). This crop is widely cultivated in Africa, Asia, Europe and Latin America, with a global production of ∼ 6 million tonnes in 2021 (Sharan et al., 2021, Bangar and Dhull, 2022, Boukid and Castellari, 2022). The increased interest in fava beans is mainly due to:

1) Agronomical advantages which allow for growth in a wide variety of environmental conditions
2) A high protein content (21–35 % w/w) with good essential amino acid profile and digestibility
3) Techno-functional features (water and fat absorption properties, emulsification, foamability, gelation etc.) of extracted protein which are suitable for a wide range of food applications (Boukid and Castellari, 2022, Sharan et al., 2021, Bangar and Dhull, 2022).

Knowledge of the impact of pre-processing and protein extraction is essential because this affects the compositional, nutritional and functional characteristics of the protein powder. Fava bean protein isolate is typically extracted through aqueous fractionation, resulting in a protein isolate with 80–95 % purity (Sharan et al., 2021). During this process, the starting material (bean flour) is first solubilized in an alkaline solution (pH 9 to 11) and insoluble materials (e.g. starch and fibre) are removed as a by-product, generally by mechanical means (Sharan et al., 2021, Bangar and Dhull, 2022, Kamani et al., 2023).The solubilized protein is subsequently precipitated at isolectric conditions by adjusting pH (pH ∼ 4 to 5) whereafter it is generally separated as a pellet, resuspended and adjusted to neutral pH before drying. Various efforts have thus far been devoted to improve the process efficacy and/or protein quality of fava beans either by optimizing process parameters or application of novel techniques (Karaca et al., 2011, Boukid and Castellari, 2022, Bangar and Dhull, 2022). However, the majority of these efforts were focused on the post-milling process (i.e., protein extraction steps), while the impact of pre-processing, mainly dehulling and milling on final protein isolate remains largely unexplored.

Preparation of the starting material (bean flour) usually involves various pre-treatments such as cleaning, sorting, soaking, dehulling and milling (Bangar and Dhull, 2022, Tiwari et al., 2011). Dehulling is a mechanical process where the seed coat (hull) is detached from the cotyledon (Bangar and Dhull, 2022, Tiwari et al., 2011). This process is an optional pre-treatment when flour is prepared from legume seeds. By removing the fibrous seed coat, antinutrients levels such as phytic acid and condensed tannins are partially reduced, and digestibility, palatability, textural and cooking qualities of legume flours are potentially improved (Tiwari et al., 2011, Sharan et al., 2021, Bangar and Dhull, 2022). Dehulling prior to milling might also be associated with a slight alteration in macronutrient composition such as an increase in flour protein content, and/or decrease in non-starch polysaccharides (Bangar and Dhull, 2022, Tiwari et al., 2011). Milling is generally the next pre-processing operation employed and is a comminution step, which provides access to the interior of seeds for subsequent extraction operations. Milling can be carried out either on whole (intact) or dehulled seed and in dry or wet form. Dry-milling is the most frequently used method, which involves directly breaking down seed particles into flour through various mechanical forces without utilizing any liquid element (Bangar & Dhull, 2022); while wet milling involves soaking and dispersing the seeds into water to soften them before wet grinding. During soaking, some antinutrients and phenolics may leach from the beans into the water, potentially increasing nutritional quality of the beans (Bangar and Dhull, 2022, Tiwari et al., 2011).

While some studies have defined the effect of the aforementioned pre-processing on legume flours, such as mucuna, lima, ukpo and common bean (Mang et al., 2015, Ibeabuchi et al., 2019, Bangar and Dhull, 2022, Okorie et al., 2013, Deshpande et al., 1982); literature relating to the effect of pre-processing on protein purity, quality and extraction efficiency is extremely limited. In addition, there is no systematic mass balance data available for the fava bean protein isolate (FPI) extraction process. Therefore, the current study aimed to determine a) whether dehulling is favourable for the production of protein isolate from fava beans and b) in the case of whole bean process, whether soaking of beans prior to wet milling is beneficial compared to dry-milling. Therefore, protein was extracted from fava beans, which underwent three different pre-processing steps:

1. Dehulling followed by dry-milling
2. Dry-milling of whole seeds
3. Soaking of whole seeds followed by wet-milling

Pre-processing operations were assessed based on the functional quality of the resultant FPI with the production yield also being taken into account.

## 2 Materials and methods

### 2.1 Materials and chemicals

Fava bean seed was obtained from Askew & Barrett Pulses Ltd., Wisbech, UK. All chemicals used in this study were of analytical grade and purchased from Merck Life Science Limited (Darmstadt, Germany).

### 2.2 Preparation of samples for protein extraction

The fava bean seeds were manually cleaned to remove foreign materials and dirt. Three different methods of preparation were used for bean samples:

(i) FP-D: the seeds were dehulled and then dry-milled into flour using a Retsch grinder (Model ZM200, Haan, Germany).
(ii) FP-WD: the whole beans (with seed coats) were directly dry-milled and passed through the mesh to obtain a uniform bean flour
(iii) FP-WW: the beans (with seed coats) were soaked in water overnight at room temperature, and then subjected to wet-milling using a blender device (TM6-1 Thermomix, Vorwerk, Germany)

In the case of (i) and (ii) flour was passed through mesh 60 (250 μm) screens to obtain a uniform bean flour. All the pre-treated samples were subjected to protein extraction.

### 2.3 Protein extraction

Protein was extracted using the alkaline solubilisation-isoelectric precipitation technique as described by Karaca et al. (2011) with slight modifications. Briefly, the prepared samples were first subjected to alkaline solubilisation (pH 11), and constantly stirred for 60 min. The samples were then centrifuged (4000 × *g*, 15 min) (Multifuge X1R Centrifuge, Thermo Scientific, Germany) to collect the supernatant. The remaining pellet was re-dispersed in distilled water at a ratio of 1:5 w/v, and re-extracted under the same conditions. Both supernatants were pooled, and adjusted to pH 4.4 to precipitate the protein. The precipitated proteins were collected by centrifugation (9000 × *g* for 20 min), and were subsequently washed, neutralized and lyophilized (Labconco, Kansas City, USA). The freeze-dried proteins were finally milled into powder, and stored for future analyses. The fava bean protein isolated from dehulled seed, whole dry-milled seed, and whole wet-milled seed were designated as FP-D, FP-WD and FP-WW, respectively. The mass yield (%) was calculated by dividing the weight of final protein powder by the weight of starting materials. The protein extraction yield was calculated from the quantities of protein extracted compared to the protein in the starting flour. Mass balance analyses were also conducted to visualize the fate of proteins in the streams generated during protein extraction.

### 2.4 Compositional analysis

Proximate composition (moisture, ash and crude fat) of fava bean flours and their derived protein isolates were analysed according to AOAC (2005). Crude protein content was estimated using the Dumas method, with a nitrogen-to-protein conversion factor of 6.25. The total starch content was determined using a starch assay kit (AA/AMG, Megazyme International, Ireland) according to the manufacturer's protocol. Total non-starchy carbohydrate was calculated by difference.

### 2.5 Protein solubility

Protein solubility (PS) was determined according to the method of Kamani et al. (2021) with slight modification. Briefly, the protein solution (at pH 7) was stirred at room temperature for 1 h, and then centrifuged at 9000 × g for 15 min. The soluble protein content of the supernatant was determined using the Dumas method (N*6.25). The PS (%) was determined as the ratio of soluble protein (in the supernatant) to the total protein content of protein powder.

### 2.6 Zeta potential (ζ-potential)

The ζ-potential value of protein samples was determined using a Zetasizer apparatus (Model Nano-ZS, Malvern Instruments Ltd., Worcestershire, UK). A protein solution (0.5 %, w/v) was freshly prepared, and then filtered using filter paper. The solution was diluted (50 times) with Milli-Q water, and then injected into a disposable measurement cell. The ζ-potential (mV) was obtained from the average value of three sequential readings using Zetasizer software (ver. 7.11).

### 2.7 Functional properties

#### 2.7.1 Water and oil absorption capacities

The water absorption capacity (WAC) and oil absorption capacity (OAC) of proteins were measured using the procedure described by Kamani et al. (2021). The protein powder was mixed with either distilled water or soybean oil and thoroughly stirred for 1 h at room temperature. The dispersion was thereafter centrifuged (4000 *× g* for 15 min, 20 °C) and the supernatant (pure water or oil) was discarded. The WAC and OAC were calculated as the amount of water or oil (g) that was absorbed per gram of protein.

#### 2.7.2 Emulsifying activity and emulsion stability

To measure the emulsifying activity index (EAI) and emulsion stability index (ESI), 5 mL of sunflower oil was mixed into a protein dispersion (0.1 % w/v in water, 15 mL) for 1 min at 15,000 rpm (Ultra turrax, model T25, IKA Instruments, Germany). Subsequently, an emulsion aliquot (50 μL) was pipetted from the bottom of the homogenate, immediately (0 min) and 10 min after homogenization, and diluted with 0.1 % (w/v) sodium dodecyl sulphate solution. After thoroughly agitating the sample with a vortex shaker, the absorbance of the diluted sample was recorded at 500 nm using a UV–Vis spectrophotometer (Spectronic Genesys 5, Rochester, USA). The EAI and ESI were calculated using the following equations (Karaca et al., 2011).

(1) $$\mathrm{EAI}\left(\frac{m^{2}}{g}\right)=\frac{2\times 2.303\times A_{500}\times DF}{L\times \emptyset \times C}$$

(2) $$ESI\left(min\right)=\frac{A_{0}}{A_{0}-A_{10}}\times t$$

where, A_500_ is the absorbance at 500 nm, DF is the dilution factor, C is the initial concentration of the sample, ∅ is the oil volume fraction, L is the optical path length, t is the time interval, and A_0_ and A_10_ are the absorbance of the diluted emulsions at time of 0 and 10 min, respectively.

#### 2.7.3 Foam capacity and stability

To determine the foaming properties, protein solution (3 % w/v in distilled water, 20 mL) was freshly prepared, and whipped at 15,000 rpm for 3 min using an IKA Ultra turrax homogenizer (model T25, IKA Instruments, Germany). The foaming capacity (FC) was measured as the increase in the percentages of foam volume upon whipping. Foaming stability (FS) was quantified as the percentage of foam volume reduction during storage times (30 – 60 min) using equations (3), (4) (Kamani et al., 2021).

(3) $$Foa\min g\;capacity\left(\%\right)=\left[\left(V_{1}-V_{0}\right)/V_{0}\right]\times 100$$

(4) $$Foa\min g\;stability\left(\%\right)=\left[\left(V_{2}-V_{0}\right)/V_{0}\right]\times 100$$

where, V_0_ is the volume of solution before whipping; V_1_ is the volume of sample solution after whipping; and V_2_ is the volume of sample solution after storage.

#### 2.7.4 Least gelation concentration

The least gelation concentration (LGC) was determined according to De Paiva Gouvêa et al. (2023) method with slight modification. Protein suspensions (5 mL) at different concentrations (2–20 % w/v in distilled water) were freshly prepared, and heated in a boiling water bath (for 1 h), followed by cooling under tap water. Afterwards, the tubes were incubated in the refrigerator for 2 h. The LGC was defined as the minimal protein concentration at which the sample did not slip after the tube was inverted (a semi-solid gel). A firm gel was deemed as a gel, which was solid with no flow.

### 2.8 Colour analysis

The differences among the colour of protein samples were monitored in a CIELAB system using a colorimeter (CR-400 Chromameter, Konica Minolta, Japan). The colorimetric parameters were measured as lightness (L*), greenness-redness (a*), and blueness-yellowness (b*). The whiteness index (WI) was measured using the equation (5) (Mang et al., 2015).

(5) $$Whiteness\;Index\;=\;\sqrt{{\left(100\;-\;L^{*}\right)}^{2}\;+\;{\left(a^{*}\right)}^{2}\;+\;{\left(b^{*}\right)}^{2}}$$

where, L*, a*, b* are lightness, redness/greenness and yellowness/blueness, respectively.

### 2.9 Statistical analyses

A completely randomized design was used for the experimental layout with three replications. All data were subjected to analysis of variance using IBM SPSS® Statistics 27 software (IBM SPSS Statistics, Chicago, USA). To determine the significant difference(s) among the experimental groups, Duncan's test was performed at a significance level of 0.05. The results were expressed as mean ± standard deviation.

## 3 Results and discussion

### 3.1 Proximate composition and yield

The proximate compositions of flours and FPI derived therefrom are shown in Table 1. The values obtained for the proximate composition of protein and flour are consistent with the previous reports (Sharan et al., 2021, Bangar and Dhull, 2022). For flours, dehulling resulted in statistically (*p* < 0.05) lower moisture and non-starch carbohydrate contents, while all other components were present in statistically higher quantities (*p* < 0.05). FP-D exhibited significantly a higher protein purity (90.12 %) (*p* < 0.05), compared to FP-WD (82.01 %). This may be due to the removal of the relatively low-protein content seed coat, which increases the protein to non-protein ratio of starting material for protein extraction, and/or better milling efficiency. The presence of the seed coat may also hinder extraction due to the adherence of protein particles to the hull fragments, which impedes appropriate detachment of protein matrix and carbohydrate (Do Carmo et al., 2020). The fava bean seed coat is a good source of phenolic compounds and fibres (Bangar & Dhull, 2022), thus it can be hypothesised that protein-fibre and/or protein–phenolic interactions may negatively affect the solubility of protein molecules under alkaline extraction conditions, leading to reduced extractability of protein in non-dehulled beans (Shen et al., 2020, Yan et al., 2020). FP-WD and FP-WW contained more carbohydrates (*p* < 0.05) than FP-D, which could be due to the co-extraction of seed coat material along with protein (Shen et al., 2020). Other studies similarly reported an increase in protein (Aryee & Nickerson, 2012; Shen et al., 2020), and reduced carbohydrate (Aryee & Nickerson, 2012; Shen et al., 2020) of the proteins extracted from different dehulled seeds. FP-WW showed the highest moisture (7.02 %) and lowest protein content (78.66 %). The soaking process may result in the absorption of water by seed components, leading to increased moisture content (Wahyuningsih et al., 2019, Ibeabuchi et al., 2019). Soaking also appeared to reduce the amount of starchy carbohydrates in the starting materials (English et al., 2019), leading to lower starch content of FP-WW (2.07 %) compared to FP-WD (7.7 %).

**Table 1:** Proximate composition (%) of fava bean flours, protein isolates and sediment by-products obtained using different methods.

Sample	Moisture	Fat	Protein	Ash	Starchy carbohydrate	Non-starchy carbohydrate
***Protein powders***						
FP-D	3.17 ± 0.15 a	1.92 ± 0.19^b^	90.12 ± 0.57^c^	4.27 ± 0.28 a	0.2 ± 0.28 a	0.31 ± 0.17 a
FP-WD	3.5 ± 0.42 a	1.58 ± 0.1 a	82.01 ± 2.85^b^	3.85 ± 0.02 a	7.7 ± 0.93^b^	1.36 ± 0.03^b^
FP-WW	7.02 ± 0.34^b^	3.16 ± 0.1^c^	78.66 ± 0.13 a	4.83 ± 0.02^b^	2.07 ± 0.49 a	4.26 ± 0.18^c^
***Alkalised sediment by-products***						
Sediment (FP-D)	5.94 ± 0.02^b^	0.31 ± 0.02^b^	2.5 ± 0.14 a	1.05 ± 0.05 a	59.83 ± 1.76^c^	30.36 ± 1.56 a
Sediment (FP-WD)	4.37 ± 0.07 a	0.69 ± 0.02^c^	7.95 ± 0.17^b^	2.54 ± 0.03^b^	36.53 ± 0.55 a	47.9 ± 0.72^c^
Sediment (FP-WW)	8.58 ± 0.05^c^	0.14 ± 0.02 a	2.3 ± 0.24 a	5.28 ± 0.02^c^	45.68 ± 1.12^b^	38.01 ± 1.35^b^
***Fava bean flours***						
Dehulled bean flour	10.22 ± 0.19 a	0.8 ± 0.01^b^	27.35 ± 0.2^b^	4.1 ± 0.07^b^	34.67 ± 0.78^b^	22.84 ± 0.95 a
Whole bean flour	12.38 ± 0.12^b^	0.68 ± 0.01 a	25.85 ± 0.13 a	3.01 ± 0.01 a	22.89 ± 0.6 a	35.17 ± 0.24^b^

a Means with different superscripts within each column are significantly different at p < 0.05. FP-D, FP-WD and FP-WW represent fava bean protein isolates extracted from dehulled seed, whole dry-milled seed and whole wet-milled seed, respectively.

**Fig. 1:**
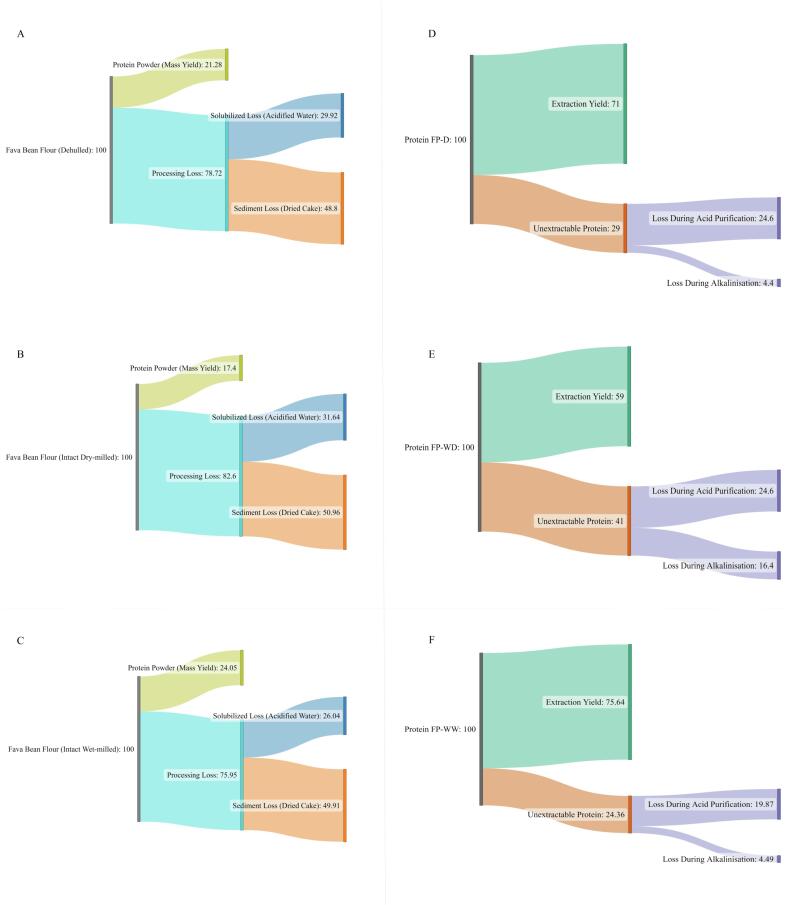
Mass balance analysis of fava bean flours (A–C) and distribution of protein components (D–E) during extraction process. FP-D, FP-WD and FP-WW represent fava bean protein isolated from dehulled seeds, whole dry-milled seeds and whole wet-milled seeds, respectively. The values were represented as weight percentages.

During the extraction process, components of the starting material (bean flour) are generally distributed into three fractions, namely, (i) the insoluble cake/sediment formed during alkalisation, (ii) the material which does not precipitate from the final acidified water suspension and (iii) the proteinaceous material which precipitates from the acidified suspension. The latter material is generally pH neutralised before drying into protein powder. Fig. 1 shows the mass and protein balances of each extraction process, with reference to the three fractions introduced above. Differences in pre-processing had remarkable effects on the distribution of the various fractions. On a mass basis, the protein powders represented 24.05, 21.28, and 17.4 % of the input flours for FP-WW, FP-D, and FP-WD, respectively (Fig. 1A-C). Although the percent weight of all alkalised sediments were similar (48–50 %), they differed significantly in composition. Comparing the contents of starchy and non-starch carbohydrates of FP-WD and FP-WW sediments with their proteins suggested an inverse relationship. That is, the higher starch content and lower level of non-starchy carbohydrates in FP-WD were inversely associated with lower starch and higher non-starchy carbohydrate of its corresponding sediment.

Fig. 1 (D-F) compares the fate of protein (based on protein content percentage) in different streams. Despite having the lowest protein content, FP-WW exhibited the highest values for extraction yields. It can be assumed that the soaking/wet milling process could facilitate solubilisation of both proteins and non-protein components. The increased solubilisation of protein likely contributed to higher extraction and mass yields. However, it seems that a greater amount of non-protein components such as carbohydrates were also simultaneously co-extracted (Bangar & Dhull, 2022), thereby reducing the protein purity. The mass and extraction yields for FP-D fall within the range of values reported in the literature (Boukid & Castellari, 2022). Dehulling can generally be expected to increase both extraction and mass yields of protein, which was in line with previous observation on hemp seed protein (Shen et al., 2020).

The yields presented in Fig. 1 (D-F) are based on starting quantities of flour, dehulled or whole. However, for the dehulled flours the fraction lost during seed coat removal may also be considered when determining yield. While the dehulled seeds in this study were purchased directly, it can be expected that ∼20 – 30 % of starting material may be lost during dehulling (Tiwari et al., 2011). Applying this loss amount (while assuming the minimum 20 % in total weight loss) to the mass yield calculation in Fig. 1, indicates that 19.5 g of protein (dry basis) can be expected from 100 g of whole seeds when using the FP-WW process. However, when seeds are dehulled prior to milling, as in the case of FP-D (with an assumption of 20 % loss in total weight) this means that 15.8 g of protein can be expected from 100 g of whole seed. This means around 28.4 % extra protein can be extracted through wet milling of whole seeds compared to dehulling combined with dry milling. In contrast, dry milling of whole seeds resulted in 6.7 % less protein compared to dry milling of dehulled seeds. Therefore, it would seem that purely on a yield maximisation basis, FP-WW is the most favourable. However, this must be balanced against the functional properties of the resultant powders, as discussed in the subsequent sections.

### 3.2 Protein solubility

Protein solubility has a direct link to functional properties; hence, it can be a good indicator of quality estimation in protein powers. Table 2 shows the solubility of extracted proteins at neutral pH. The protein extracted from whole seeds (FP-WD and FP-WW) had noticeably lower protein solubility (PS) than FP-D. It could be hypothesized that the interaction of protein with remaining non-protein components, in particular phenolic compounds co-extracted from the seed coat, resulted in lower PS. Phenols possess a binding affinity to proteins, leading to the formation of insoluble phenol–protein complexes (Sęczyk et al., 2019; Fernando, 2020). In this context, Fernando (2020) highlighted that binding of phenolic compounds to hydrophilic groups of protein molecules can change the surface charge thereby negatively affecting PS. Variations in protein composition, molecular weights, solubility level of protein subunits (legumin and vicilin) and hydrophobic/hydrophilic balance could be other reasons for the observed differences. It must be noted that PS can also be influenced by a range of environmental factors (pH, temperature), as well as the type and parameters of processes applied (e.g. extraction methods, drying methods) (Karaca et al., 2011, Vogelsang-O’Dwyer et al., 2020, Viana and English, 2022, Boukid and Castellari, 2022). In literature, a wide range of PS (from 25 to 85 % at neutral pH) was reported for the solubility of fava bean protein isolated through isoelectric precipitation (Vogelsang-O’Dwyer et al., 2020, Karaca et al., 2011, Chiremba et al., 2018, Boukid and Castellari, 2022). The PS values obtained in the present study were consistent with the reported range. Overall, the above results suggest that dehulling could lead to superior PS in fava bean. Such enhancement is advantageous in formulating plant-based protein beverages, where good solubility is often required.

**Table 2:** Protein solubility (PS), zeta potential (ζ-potential), foaming capacity (FC), and foam stability (FS) of fava bean proteins extracted from whole and dehulled seeds a

Sample	PS (%)	ζ-potential (mV)	FC (%)	FS (%) − 30 min	FS (%) − 60 min
FP-D	64.33 ± 2.38^c^	− 31.46 ± 2.31^b^	38.75 ± 1.76^b^	33.75 ± 1.76^b^	26.75 ± 0.35 a
FP-WD	54.85 ± 3.98^b^	− 39.16 ± 2.54 a	30.25 ± 0.34 a	26.5 ± 1.4 a	25.5 ± 0.7 a
FP-WW	45.26 ± 3.55 a	− 39.52 ± 0.67 a	34 ± 1.41 a	32.75 ± 0.35^b^	25.25 ± 0.3 a

a Means with different superscripts within each column are significantly different at p < 0.05. FP-D, FP-WD and FP-WW represent fava bean protein isolates extracted from dehulled seed, whole dry-milled seed and whole wet-milled seed, respectively.

### 3.3 Zeta potential (ζ-potential)

All protein samples carried a negative charge at neutral pH (Table 2). This was due to the fact that the protein samples were analysed at above their isoelectric points. FP-D exhibited a relatively high ζ-potential value (-31.46 mV), which was within the reported range (-28 to −34 mV) in the literature (Vogelsang-O’Dwyer et al., 2020, Żmudziński et al., 2021, Keivaninahr et al., 2021). FP-WW and FP-WD showed similar ζ-potential values. As presented in Table 1, FP-WW and FP-WD had significantly lower protein content than FP-D, however, their ζ-potential magnitudes were surprisingly found to be higher (more negative). Two potential reasons can be given for the observed differences: (i) a difference in the amino acid composition of protein samples; and (ii) the presence of phenolic compounds in protein ingredients, which contribute to ζ-potential value (Shen et al., 2020, Yan et al., 2020, Fernando, 2020). In this context, Shen et al. (2020) observed a slight difference in the ζ-potential magnitude of proteins isolated from dehulled and non-dehulled hemp seed, which was attributed to the differences in amino acid composition. Yan et al. (2020) pointed out that the presence of phenolic compounds may also increase ζ-potential value in *Cinnamomum camphora* seed kernel protein. In addition to the above reasons, fibrous material (present in legume seed coats) may also have a certain ζ-potential value that contributes to the overall shift of charge of solution (Kamani et al., 2019). Therefore, it could be inferred that the protein content is not the only contributing factor to ζ-potential of a protein powder. Apart from the composition of protein and non-protein compounds, factors such as pH, conductivity, seed genotype, extraction technique, and presence of additives might also cause significant variation in the ζ-potential of proteins (Karaca et al., 2011, Martinez et al., 2016, Keivaninahr et al., 2021, Kamani et al., 2019). Generally, a solution with ζ-potential more than + 30 mV or lower than −30 mV is considered a stable suspension (Kamani et al., 2019). Therefore, the above results indicate the materials generated in this study are potentially suitable for making various food dispersion systems. However, further understanding is still required to specifically unravel how non-protein ingredients can bond with protein molecules and alter their ζ-potential within the dispersion system.

### 3.4 Water and oil absorption capacities

**Fig. 2:**
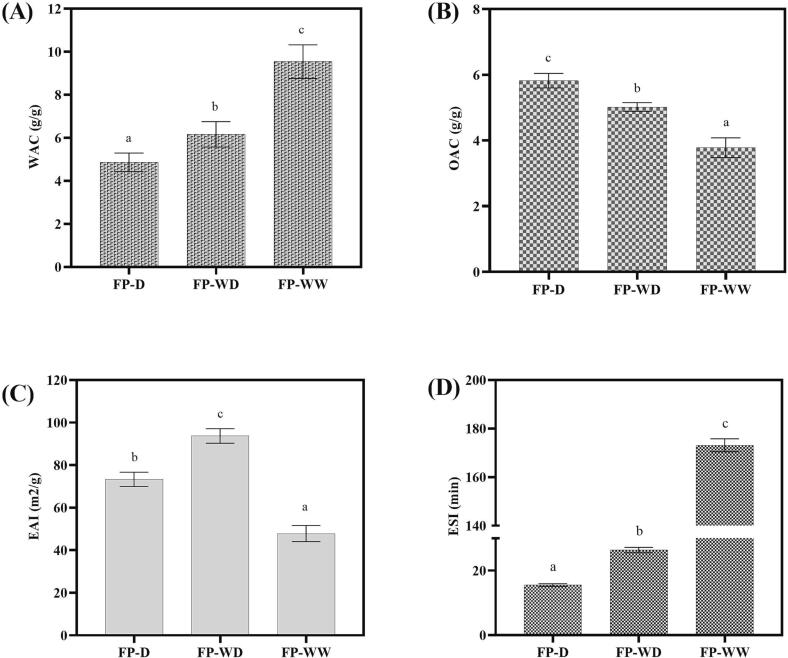
Functional characteristics of fava bean proteins extracted from whole and dehulled seeds. Means with different superscripts are significantly different at p < 0.05. FP-D, FP-WD and FP-WW represent fava bean protein isolates extracted from dehulled seed, whole dry-milled seed and whole wet-milled seed, respectively. WAC: Water absorption capacity. OAC: Oil absorption capacity. EAI: Emulsifying activity index. ESI: Emulsion stability index.

Water absorption capacity (WAC) and oil absorption capacity (OAC) indicate the ability of a protein powder to physically retain water or oil molecules when subjected to external stress such as centrifugation force (Zhang et al., 2021, Mang et al., 2015). Evaluation of such interactions allows us to understand the capability of a protein powder in preventing fluid leakage during food processing and storage. The WAC of proteins is shown in Fig. 2A. The WAC of FP-D (4.86 g/g) was comparable to the figured reported by Keivaninahr et al., (2021), but higher than the values recorded by De Paiva Gouvêa et al., (2023) and Żmudziński et al., (2021). This difference could be due to variations in pre-processing, extraction protocols an also protein composition (Keivaninahr et al., 2021, Tiwari et al., 2011). However, dehulling significantly (p < 0.05) reduced WAC of protein samples, compared to FP-WD and FP-WW. A similar reduction was reported for WAC of pre-soak yellow eye beans (Viana et al., 2022), lima beans (Ibeabuchi et al., 2019), and horse gram flours (Pal et al., 2016) as a consequence of dehulling. This could be explained by the presence of a larger amount of water-binding matrix components (starch and fibre) in FP-WD and FP-WW (Table 1), contributing to greater water retention in whole seed protein powders (Fernando, 2020; Viana et al., 2022).

As shown in Fig. 2B, FP-D exhibited the highest OAC (5.82 g/g), followed by FP-WD (5.02 g/g), and FP-WW (3.78 g/g). This trend could be linked to their protein contents (Table 1), where a higher level of protein allows greater entrapment of oil molecules. Similar trends were found in OAC of sorrel seed (Ayo-Omogie & Osanbikan, 2019) and boiled Mucuna bean (Mang et al., 2015) where dehulling increased the protein contents in the seed flours. The value obtained for OAC of FP-D is consistent with the value previously reported by Eckert et al. (2019), but higher than those reported by other studies (Keivaninahr et al., 2021, De Paiva Gouvêa et al., 2023, Martinez et al., 2016). Apart from process conditions and protein concentration, WAC and OAC can be affected by several factors such as protein source, pH, protein charge, temperature, protein particle size, hydrophobicity-hydrophilicity level and presence of non-protein components (e.g., salts, lipids or polysaccharides) (Zhang et al., 2021, Keivaninahr et al., 2021). WAC and OAC are of great importance as they are directly linked to texture, flavour retention and mouthfeel of food products (Bangar & Dhull, 2022). Interestingly, the present study demonstrates suitable WAC and OAC for all protein groups, suggesting their potential application for food formulations like bakery, emulsified meat or fried products, where water/oil absorption ability is an important consideration.

### 3.5 Emulsifying activity and emulsion stability indexes

Emulsions are inherently unstable due to interfacial tension between oil and water phases. Proteins are widely used as emulsifiers to stabilize emulsions by lowering this interfacial tension. During the homogenization of oil, proteins can migrate to the oil–water interface and reconfigure so that their polar and non-polar amino acid residues are orientated toward the water and lipid phases, respectively. This results in the development of a protective layer around lipid droplets, which helps to prevent phase separation (Zhang et al., 2021). As shown in Fig. 2C, FP-WD showed the highest EAI amongst all samples. This could be ascribed to the higher content of starch in FP-WD, which might synergistically increase EAI along with protein molecules (Hojilla-Evangelista & Evangelista, 2017). Dehulling induced a significant decrease in the emulsifying activity index (EAI) of dry-milled fava bean protein, which was in agreement with previous reports on coriander protein extracts (Hojilla‐Evangelista & Evangelista, 2017) and black bean protein (Fernando, 2020). The EAI recorded in the present study was higher than previous reports (Karaca et al., 2011, Eckert et al., 2019, Nivala et al., 2021). This variation could be due to differences in extraction method, composition, physiochemical and molecular characteristics (e.g., solubility, molecular size, structural flexibility, surface charge and hydrophilic-hydrophobic amino acids ratio) of protein (Tiwari et al., 2011; Fernando, 2020; Möller, 2021; Bangar & Dhull, 2022). EAI may be also affected by temperature, ionic strength, pH and the viscosity of aqueous phase, and the methodology used for EAI measurement (Zhang et al., 2021, Nivala et al., 2021, Fernando, 2020). Therefore, the comparison between EAI values of the present study with literature is challenging.

Emulsion stability index (ESI) is an indicator that monitors the decrease in turbidity of diluted emulsion over a defined time (Tiwari et al., 2011, Karaca et al., 2011). While the ESI value obtained for the dehulled sample (15.53 min) was consistent with ESI (17.6 min) from a previous study on native fava bean protein isolate prepared with a similar method, such comparisons should be interpreted catiously as laboratory specific experimental conditions (e.g. initial emulsion formation conditions) may affect the final result (Nivala et al., 2021). As shown in Fig. 2D, a protein with high EAI may not always present high stability. Compared to FP-D, FP-WD and FP-WW produced more stable emulsions. In accordance with the present results, other researchers documented the reducing effect of dehulling on emulsion stability of plant-based protein extract and flours (Deshpande et al., 1982; Hojilla‐Evangelista & Evangelista, 2017; Ayo-Omogie & Osanbikan, 2019). The ability of a protein ingredient to form and stabilize emulsions plays an important role in developing food products. Given the relatively high emulsification value obtained in the present study, fava bean protein can serve as a promising plant-based emulsifier agent in the production of emulsified foods like mayonnaise, sausage, etc.

### 3.6 Foam capacity and stability

Foam is a two-phase mixture in which the gaseous phase is surrounded by a liquid continuous phase. Proteins are surface active, amphiphilic molecules and can stabilise foams by reducing surface interfacial tension in aerated liquids. Foamability of a protein is typically expressed as foaming capacity (FC), which represents the increase in relative volume of a system after whipping (Tiwari et al., 2011). Depending on the extraction method technique/conditions, fava bean protein isolate may possess a broad range of FC (from 15 to 77 %) (Boukid and Castellari, 2022, Chiremba et al., 2018). The present FC results (Table 2) fall within the reported range in the literature. FP-D showed significantly (*p* < 0.05) higher FC than the protein extracted from intact seeds (FP-WD and FP-WW), indicating that FPI from dehulled flours may be superior for foaming applications. This observation is consistent with previous studies which found dehulling to have a beneficial effect on foam expansion in systems containing proteins from coriander (Hojilla‐Evangelista & Evangelista, 2017) and pulse seeds (Fernando et al., 2021). This higher FC in FP-D could be attributed to its higher powder protein concentration (Table 1). Solubility of protein is another contributing factor to FC, as soluble proteins are mainly involved in foam formation. As shown in Table 2, protein extracted from intact seeds (FP-WD and FP-WW) had considerably lower solubility levels than FP-D. This could be due to the presence of phenolic compounds (co-extracted from seed coat), which can block the hydrophilic groups of protein, and decrease solubility, ultimately leading to a reduced foamability of protein (Fernando, 2020). A comparable FC (*p* > 0.05) was found between soaked (FP-WW) and coarse ground proteins (FP-WD). Despite the lower protein content and solubility of FP-WD and FP-WW, their FC was still appreciable. This probably could be due presence of non-protein components with distinct foaming abilities, in particular saponins, which could be carried over with the protein (from seed coat) during alkaline extraction (Tiwari et al., 2011, Kamani et al., 2021).

Foams are unstable colloidal systems and disintegrate during storage. The ability of protein to stabilize foams depends on the protein–protein interactions, and may not essentially be linked to their capability to create a foam (Do Carmo et al., 2020). In other words, FC is an indication of air uptake and is not necessarily a measure of its stability. As is shown in Table 2, a decreasing trend, with various degrees of instability, was observed in the foam volume of all proteins over the incubation period (30 and 60 min). After 30 min incubation, FP-D showed a higher FS value than FP-WD, which was consistent with previous reports (Hojilla‐Evangelista & Evangelista, 2017; Fernando, 2020; Fernando, 2021). This was probably due to the higher protein content and greater solubility of FP-D. Another factor may be that higher protein solubility is generally linked with increased viscosity of system, which facilitates the formation of a multilayer cohesive protein film at the air–water interface of the foams, with improved FS (Bangar & Dhull, 2022). Despite the lower protein content and solubility level of FP-WW, this sample produced more stable foams (at 30 min) than FP-WD. This result possibly reflects the influence of the remaining non-protein constituents in the protein extract. For instance, some complex carbohydrates located in the seed coat (with hydrophilic nature) might be co-extracted during the extraction process, and increase the viscosity of system, leading to enhanced FS by preventing collapse of foam bubbles (Deshpande et al., 1982, Tiwari et al., 2011). This hypothesis can be supported by the non-starchy carbohydrate data shown in Table 1. Apart from the above factors, a wide range of parameters including protein surface charge, hydrophobicity, pH, molecular size and the extent of protein denaturation during processing can largely change FC and FS protein powders (Tiwari et al., 2011; Fernando, 2020; Bangar & Dhull, 2022). The above foaming outcome suggests that all fava bean proteins have promising foamability, which can be used to create a unique texture in aerated food systems where overrun/foam formation is desirable (e.g., meringues. ice cream, whipped toppings and cream). Dehulling may be considered to somewhat improve foaming properties, however any benefit may disappear over longer storage times.

### 3.7 Least gelation concentration

**Fig. 3:**
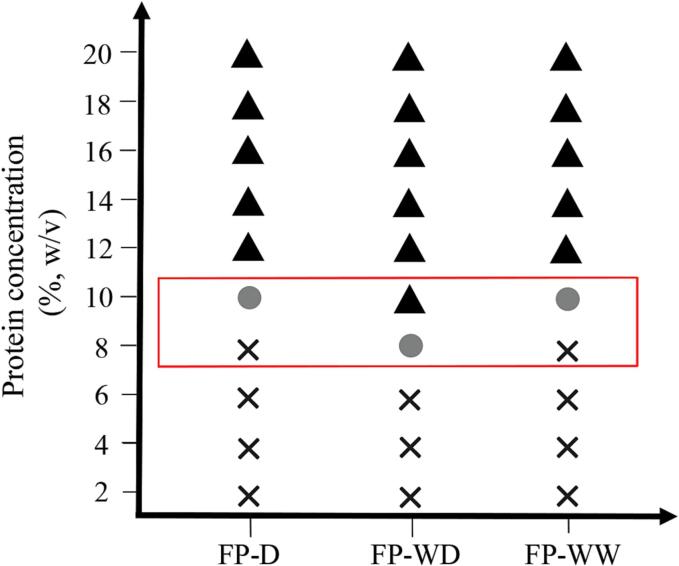
Mapping of least gelation concentration (LGC) of fava bean proteins as a function of protein concentration. (**×**),  and  indicate no gelation, gelation and firm gelation, respectively. FP-D, FP-WD and FP-WW represent fava bean protein isolates extracted from dehulled seed, whole dry-milled seed and whole wet-milled seed, respectively.

The least gelation concentration (LGC) is a measure of the gelation capacity of food proteins. Proteins with lower LGC values suggest a better capacity to form self-supporting gels (Bangar & Dhull, 2022; Fernando, 2020; Tiwari et al., 2011). Fig. 3 shows the LGC of fava bean proteins, which ranged from 8 to 10 %. No gels were formed from any protein powders at 2 to 6 % concentration, whereas firm gels were observed at the concentration above 12 % for all protein samples. The LGC value obtained for FP-D was in agreement with the study of Nivala et al. (2021) who reported gel formation of fava bean protein isolate (at 10 % concentration) under heat and acidic conditions. FP-WD showed a marginally lower LGC value (8 %) than FP-D. This difference could be explained by the fact that the gel-forming capacity of protein is not only affected by protein concentration but also by the presence of non-protein components such as starch and fibrous components (Tiwari et al., 2011, Pelgrom et al., 2015; Fernando, 2020; Boukid & Castellari, 2022). In this context, other researchers also emphasized the important role of gel-forming carbohydrates in greater gelation ability and characteristics of plant proteins (Vogelsang-O’Dwyer et al., 2020, Boukid and Castellari, 2022, De Paiva Gouvêa et al., 2023). For instance, fava bean starch can be gelatinized upon heat denaturation of protein, resulting in higher viscosity within the system, thereby contributing to gel formation (Pelgrom et al., 2015, Bangar and Dhull, 2022).

Interestingly, despite the lower protein content of FP-WW compared to FP-D, its LGC was found to be similar. This could be explained by the presence of non-starchy carbohydrates in this sample that could contribute to protein gelation. Partial absorption of water and reducing the available water within the system by fibre, may improve LGC. This is consistent with the high WAC of FP-WW as reported in Fig. 2A. That being said, the gelation of legume proteins largely depends on their amino acid profile and subunits of proteins, seed variety, ionic strength, pH, heating conditions and method applied for protein processing (Tiwari et al., 2011, Pelgrom et al., 2015; Fernando, 2020; Zhang et al., 2021, De Paiva Gouvêa et al., 2023). Protein ingredients with good gel-forming ability can be used as texture modifiers to improve the organoleptic properties of food systems. From the above results, fava bean protein showed a promising LGC, and therefore it is potentially suitable for a broad range of food applications (e.g., glass noodles, jelly-type desserts, pudding, meat products) which require high levels of gelation. However, this conclusion still needs further investigation to deeply understand its molecular mechanism (protein–protein interaction and protein interaction with non-protein components) within heat-induced gel networks, and identify optimum processing conditions based on the target application. The above observation also indicated that FP-WW may potentially be useful as an alternative gelling agent in foods where very high protein purity is not required.

### 3.8 Colour

**Fig. 4:**
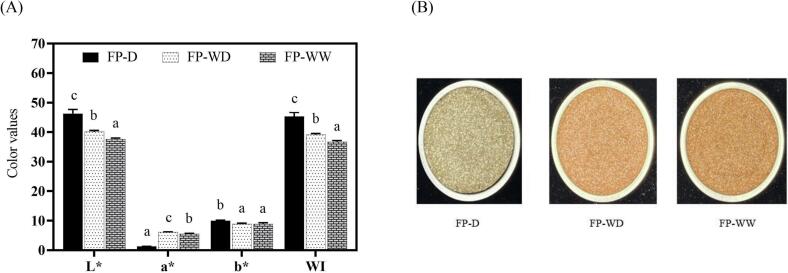
The colorimetric parameters (L*, a* and b*, WI) (A) and the appearance (B) of fava bean protein powders obtained from whole and dehulled seeds. Means with different superscripts are significantly different at p < 0.05. FP-D, FP-WD and FP-WW represent fava bean protein isolates extracted from dehulled seed, whole dry-milled seed and whole wet-milled seed, respectively. L*, a*, b* and WI indicate lightness, redness/greenness, yellowness/blueness and whiteness index, respectively.

The colour of protein powder plays a key role in customer/consumer choices. As depicted in Fig. 4, all protein samples had a dark appearance. This was mainly due to the oxidization of polyphenol compounds during alkaline extraction, leading to the generation of dark colour (Kamani et al., 2023). FP-D exhibited a higher L* and b*, but lower a* compared to FP-WD and FP-WW. As expected, whiteness index (WI) of FP-D was also found to be significantly higher than other groups (*p* < 0.05). The higher lightness/whiteness of FP-D could be ascribed to its lower content of phenolic compounds (Shen et al., 2020; Fernando, 2020). The fava bean seed coat is usually beige or light brown colour– largely as a result of high quantities of phenolic compounds. Similar colours were noticed for FP-WD and FP-WW (Fig. 4B), which may be ascribed to the presence of seed coat during protein solubilisation at alkaline conditions, resulting in a higher presence of co-extracting phenols. Despite the visual appearance of FP-WD and FP-WW in Fig. 4B, FP-WD showed slightly higher L* and WI compared to FP-WW. This might be due to the higher content of starch in this sample, contributing to lighter surface colour. From a commercial standpoint, the dark colour of protein ingredients can contribute to discoloration in finished products, resulting in lower consumer acceptability. Hence, attention should be paid to further improve its market potential through decolourization processing.

## 4 Conclusion

The impact of pre-processing variables (dehulling and milling) on compositional changes and techno-functional properties of fava bean protein was reported for the first time. Dehulling caused remarkable differences in the yield, chemical composition and functionality of protein isolates. Compared to protein extracted from dry-milled whole seed flour (FP-WD), protein extracted from dehulled flour (FP-D) resulted in significantly higher yield, protein purity, OAC, PS and foamablity (*p* < 0.05). However, the commonly applied method of soaking whole beans prior to wet milling and protein extraction (FP-WW) was superior in terms of mass yields, extraction yields, WAC, and ζ-potential in comparison with other samples. Overall, the results indicated that while all pre-processing variations resulted in protein powders with potential for use in food formulations, dehulling is an important step where high purity and solubility are required. Omitting the dehulling step may offer superiority in terms of gelation, emulsification, and WAC. However, the presence of impurities (mainly carbohydrates) in such protein powders may limit applications, particularly where high protein purity is a key requirement. From a nutritional perspective, further research work is underway to provide deeper insight into the amino acid profile, digestibility, and antinutritional levels of produced protein samples.

## CRediT authorship contribution statement

**Mohammad Hassan Kamani:** Writing – original draft, Investigation, Methodology, Conceptualization. **Jianlei Liu:** Investigation, Methodology. **Sinead M. Fitzsimons:** Project administration, Supervision, Writing – review & editing. **Mark A. Fenelon:** Project administration, Resources, Supervision, Writing – review & editing. **Eoin G. Murphy:** Conceptualization, Investigation, Methodology, Project administration, Supervision, Writing – review & editing.

## Declaration of competing interest

The authors declare that they have no known competing financial interests or personal relationships that could have appeared to influence the work reported in this paper.

## Data Availability

Data will be made available on request.
